# How long does it take community mental health team staff to suspect autistic spectrum disorder?

**DOI:** 10.1192/bjo.2021.146

**Published:** 2021-06-18

**Authors:** Kirsty Knight, Ian Ellison-Wright

**Affiliations:** Fountain Way Hospital

## Abstract

**Aims:**

We wanted to discover the time delay between the initial assessment of service users referred to a Community Mental Health Team (CMHT) and suspicion that they had an Autistic Spectrum Disorder (ASD). We wanted to know whether early use of a screening questionnaire could significantly reduce this delay.

**Background:**

About 1% of the UK population have ASD and the rate is higher among service users within CMHTs. Although CMHT staff are trained to recognize service users with ASD, often the diagnosis is only suspected when service users do not make progress with standard treatment. Early recognition of ASD informs a treatment pathway individualised for people with ASD. Brief screening instruments for ASD can help clinicians decide whether to refer someone for a full diagnostic assessment. The fifty question Autism Questionnaire (AQ50) and ten question Autism Questionnaire (AQ10) both perform well as a screen for ASD.

**Method:**

All referrals from two adult CMHTs to a specialist Wiltshire Autism Diagnostic service (WADS) over a 2.5 year period were ascertained from a referral database. 24 service users referred from the CMHTs were identified. We determined from their records: (A) overall time between initial CMHT appointment and referral to WADS, (B) time between initial CMHT appointment and screening test (when used), (C) time between screening test and referral to WADS.

**Result:**

For all 24 cases, the average time between initial CMHT appointment and referral to WADS was 186 days. 18 of the 24 service users completed a screening questionnaire prior to WADS referral (AQ10 or AQ50 or both); 16 of these had positive screening tests. The average time between initial CMHT appointment and use of screening test was 164 days. The average time between screening test use and referral to WADS was 32 days.

**Conclusion:**

Our results demonstrated the average time taken from CMHT staff first seeing a patient to suspecting ASD and referring to a specialist diagnostic team was about 6 months. However, after a screening questionnaire had taken place, the time to referral was only around one month. We propose that screening is considered at an earlier opportunity; ideally during (or prior to) the first appointment with the CMHT in order to reduce the time before a referral to a specialist diagnostic team is made. This would enable treatment in a care pathway which incorporates the diagnosis of ASD at an earlier stage.

